# Pulsed field ablation of superior vena cava in paroxysmal atrial fibrillation: a case report

**DOI:** 10.3389/fcvm.2023.1211674

**Published:** 2023-06-30

**Authors:** Yirao Tao, Yang Zhou, Xuerong Sun, Wenkai Liao, Yanjiang Wang, Liang Shi, Xingpeng Liu

**Affiliations:** Heart Center, Beijing Chaoyang Hospital, Capital Medical University, Beijing, China

**Keywords:** pulsed field ablation, paroxysmal atrial fibrillation, superior vena cava, arrhythmogenesis, radiofrequency ablation

## Abstract

Paroxysmal atrial fibrillation originates most commonly in the pulmonary veins. However, the superior vena cava has proved to be arrhythmogenic in some cases. Pulsed field ablation, an emerging ablation technology, selectively affects myocardial tissue. Herein, we present a case of paroxysmal atrial fibrillation in a 64-year-old man who was admitted to our hospital for pulsed field ablation. The tachycardia was recurrent despite four successful pulmonary vein isolations. The superior vena cava was determined to be involved in arrhythmogenesis. The atrial fibrillation terminated immediately after the pulsed field ablation discharge at the superior vena cava.

## Introduction

Atrial fibrillation is the most common sustained arrhythmia, and pulmonary vein isolation is the cornerstone for ablation of paroxysmal atrial fibrillation. Pulmonary vein isolation is commonly performed using cryoballoon or radiofrequency ablation. The “Fire and Ice” trial demonstrated that radiofrequency ablation and cryoballoon ablation were comparable with respect to efficacy and safety in paroxysmal atrial fibrillation ablation (1). Pulsed field ablation is an emerging ablation technique that selectively ablates myocardial tissue. Theoretically, the application of pulsed field ablation to paroxysmal atrial fibrillation is safer because it avoids damage to collateral tissues. Furthermore, the procedural and fluoroscopy time are shorter in pulsed field ablation than in radiofrequency ablation.

The superior vena cava is a common location of non-pulmonary vein foci of atrial fibrillation (2). In selected patients in whom the superior vena cava is identified as a trigger for atrial fibrillation, superior vena cava ablation significantly reduces atrial fibrillation recurrence (3). Pulsed field ablation has excellent effectiveness and safety in isolating pulmonary veins (4). However, pulsed field ablation applied to the superior vena cava in paroxysmal atrial fibrillation has not been reported. Herein, we report a case in which paroxysmal atrial fibrillation terminated after the discharge of pulsed field ablation at the superior vena cava.

## Case presentation

A 64-year-old man with a two-year history of paroxysmal atrial fibrillation was admitted to our facility for palpitations. The tachycardia was recurrent despite four successful pulmonary vein isolations. His electrocardiogram at admission displayed sinus rhythm, whereas the electrocardiogram during palpitations displayed atrial fibrillation. His physical examination findings and blood test results were unremarkable. Transesophageal echocardiography revealed no thrombosis of the left atrial appendage. The left ventricular ejection fraction was 69% and the left atrial diameter was 40 mm. The left atrial volume was 112 ml according to a three-dimensional electroanatomical map. With the patient's informed consent, pulsed field ablation was performed under general anesthesia.

**Figure 1:**
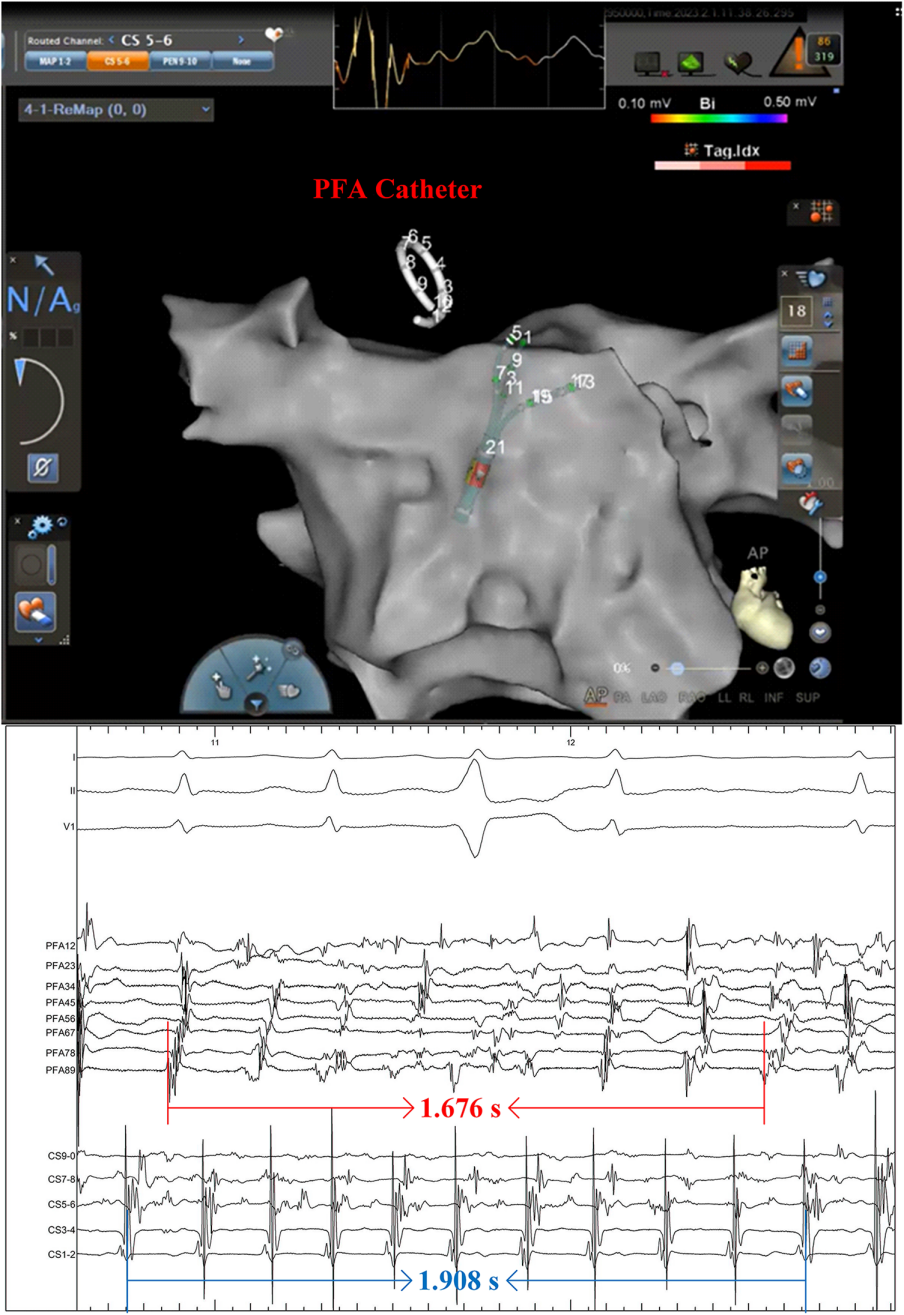
A pulsed field ablation catheter was positioned within the superior vena cava, revealing the disorganized electrical activity. The intracardiac electrocardiogram was recorded at a speed of 100 mm/s.

**Figure 2:**
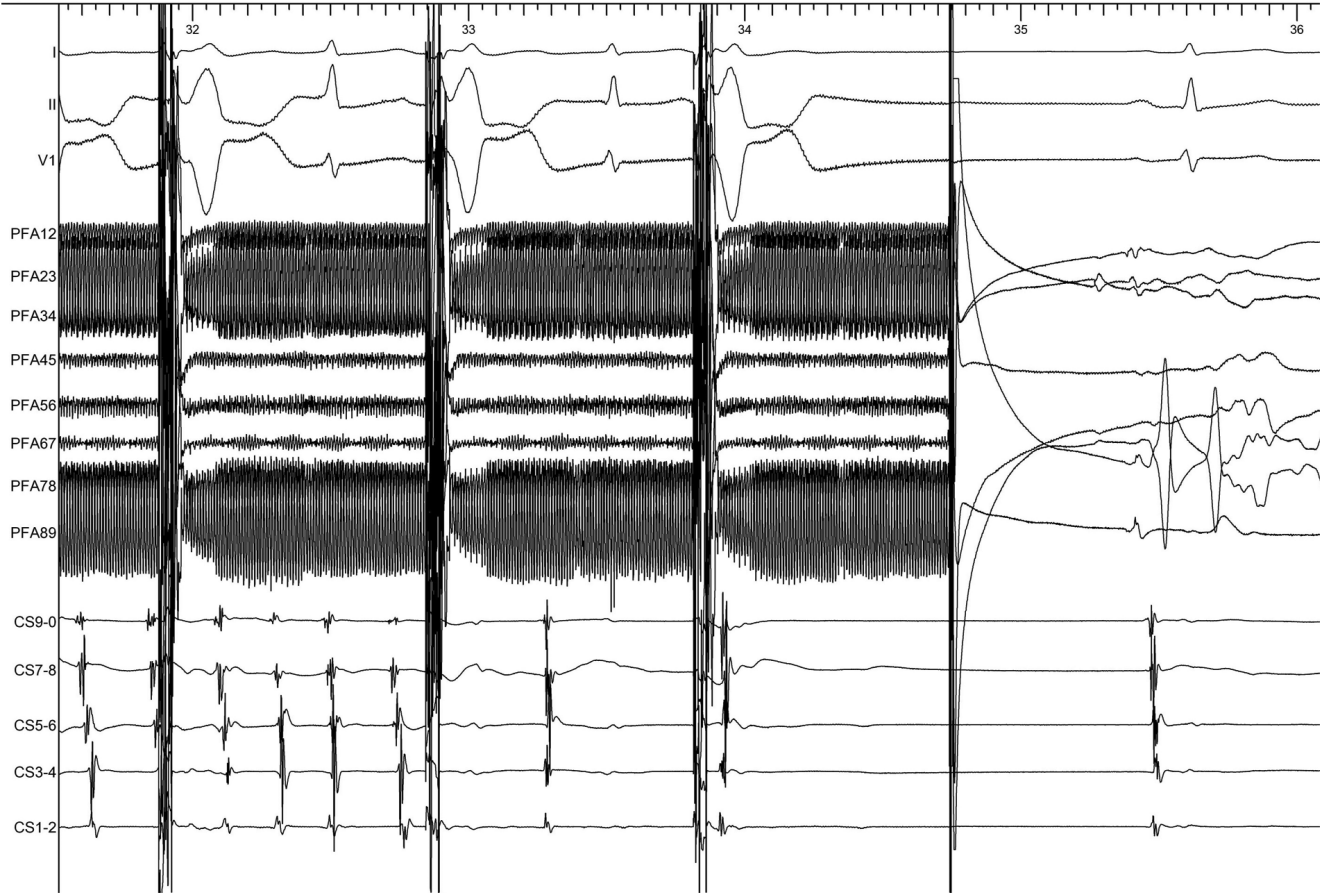
The atrial fibrillation terminated after the pulsed field ablation catheter discharged at the superior vena cava. The electrocardiogram was recorded at a speed of 100 mm/s.

**Figure 3:**
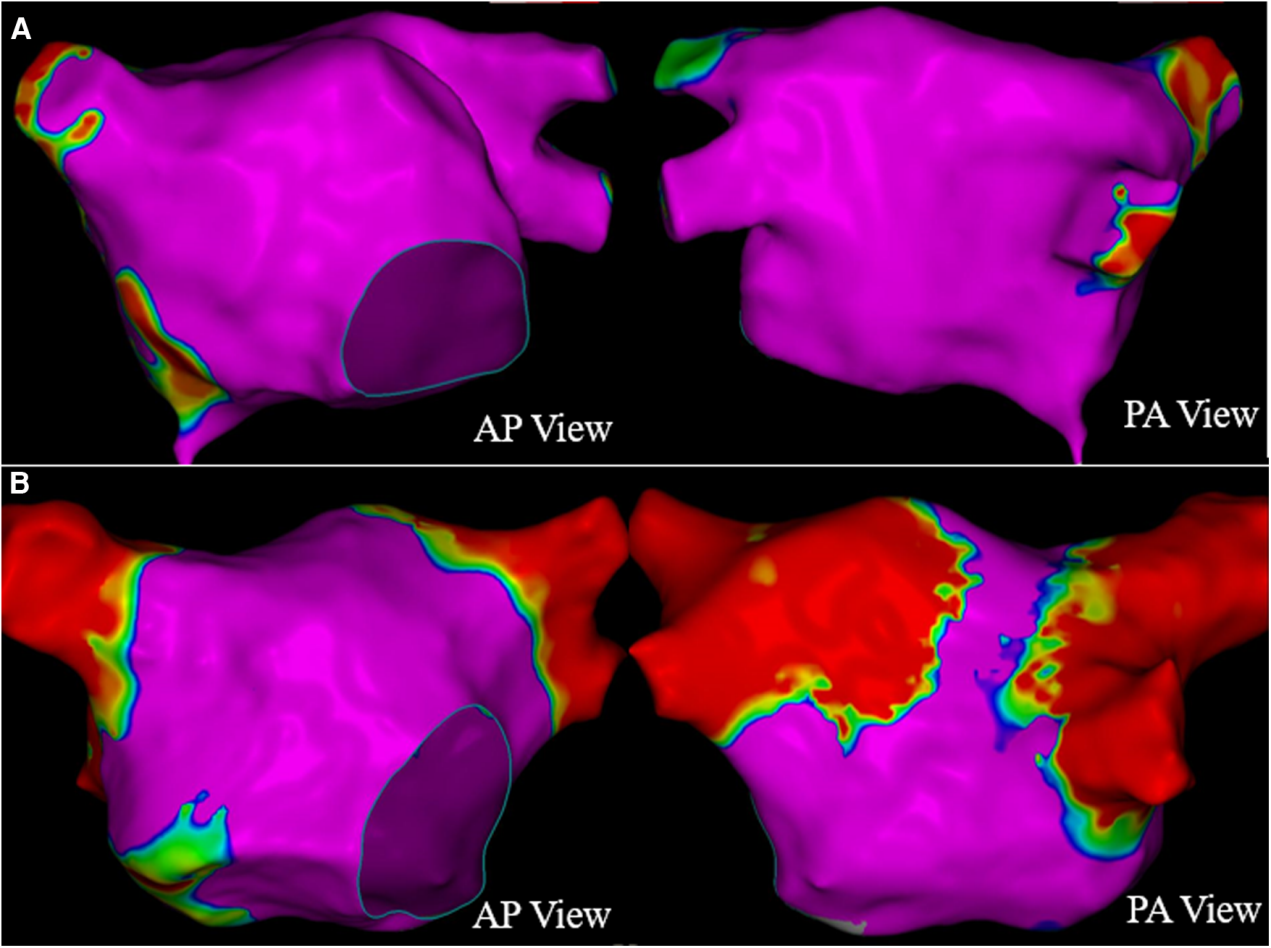
The left atrial voltage map pre- (**A**) and post- (**B**) pulsed field ablation.

The patient presented sinus rhythm at the beginning of the procedure. However, atrial fibrillation recurred during left atrial mapping using a PentaRay catheter (Biosense Webster, Inc., Irvine, CA, USA). After all pulmonary veins were successfully isolated using an 8-F circular pulsed field ablation catheter (Shineyo Medical, Shanghai, China), atrial fibrillation persisted. The circular pulsed field ablation catheter was then placed within the superior vena cava, revealing some brief fractionated potentials and a local atrial fibrillation cycle length shorter than that in the coronary sinus (Figure 1). The atrial fibrillation terminated after a pulsed field ablation catheter discharge at the superior vena cava (biphasic, 1,400 V) (Figure 2). The left atrial voltage map pre- and post-pulsed field ablation is shown in Figure 3. The total procedure time was 2 h with a superior vena cava ablation time of 20 s.

According to the monitoring parameters, such as heart rate, cerebrovascular risk assessment, and diaphragm mobility, there were no peri-procedural complications. The patient was free of atrial fibrillation at the three-month follow-up.

## Discussion

To the best of our knowledge, this is the first reported case of pulsed field ablation of the superior vena cava in a patient with paroxysmal atrial fibrillation. Pulsed field ablation appears to be safe and effective for superior vena cava isolation.

Pulsed field ablation is a novel and promising ablation technique in which ultrafast electrical fields (in seconds) are applied to myocardial tissue (5). In a 2019 human trial, Reddy et al. (6) first demonstrated that pulsed field ablation has excellent tissue selectivity and preferentially ablates myocardial tissue. Furthermore, pulsed field ablation can rapidly and durably isolate pulmonary veins (6). Therefore, the pulsed field ablation was deemed to be a promising method in pulmonary vein isolation.

Atrial fibrillation is most frequently initiated by the pulmonary veins. However, 10% to 20% of atrial fibrillation originates from extra-pulmonary vein locations, such as the superior vena cava, posterior left atrial wall, Marshall ligament, crista terminalis, coronary sinus ostium, and left atrial appendage (7–9). Among non-pulmonary vein triggers, superior vena cava origins account for approximately 37%, next to the posterior left atrial wall (7). Superior vena cava isolation is commonly performed using radiofrequency energy. However, stenosis of the superior vena cava and injury to the sinoatrial node and phrenic nerve are potential complications of radiofrequency ablation of the superior vena cava (10–12). Furthermore, as compared with pulsed field ablation, superior vena cava isolation using radiofrequency has a more complex workflow because of point-by-point ablation and tagging the location of the phrenic nerve on the three-dimensional map. In contrast, pulsed field ablation has exceptional tissue selectivity and no effect on the phrenic nerve (13). Moreover, pulsed field ablation delivers electric pulses of very short duration that result in irreversible tissue injury. In the present case, the superior vena cava was isolated in 20 s, as compared with an average of 7.8 min for radiofrequency ablation and 36.9 s for cryoballoon ablation in previous studies (14, 15).

The feasibility and safety of pulsed field ablation of the superior vena cava were demonstrated in a previous animal trial (16). We preliminarily demonstrated that pulsed field ablation is effective and safe for superior vena cava isolation in patients with atrial fibrillation. Regrettably, anatomical mapping of the right atrium and superior vena cava were not performed.

## Conclusion

We have reported a case of successful superior vena cava isolation using pulsed field ablation in a patient with atrial fibrillation. Pulsed field ablation is an alternative method to isolate the arrhythmogenic superior vena cava, with potential advantages of shorter procedure time and fewer complications than radiofrequency.

## Data Availability

The original contributions presented in the study are included in the article, further inquiries can be directed to the corresponding author.
